# Fitness of Herbicide-Resistant Weeds: Current Knowledge and Implications for Management

**DOI:** 10.3390/plants8110469

**Published:** 2019-11-01

**Authors:** Martin M. Vila-Aiub

**Affiliations:** 1 IFEVA, CONICET, Department of Ecology, Faculty of Agronomy, University of Buenos Aires (UBA), Buenos Aires 1417, Argentina; vila@ifeva.edu.ar; 2 School of Agriculture & Environment, University of Western Australia (UWA), Crawley, WA 6009, Australia

**Keywords:** resistance mutation, fitness benefit, fitness cost, resistance management

## Abstract

Herbicide resistance is the ultimate evidence of the extraordinary capacity of weeds to evolve under stressful conditions. Despite the extraordinary plant fitness advantage endowed by herbicide resistance mutations in agroecosystems under herbicide selection, resistance mutations are predicted to exhibit an adaptation cost (i.e., fitness cost), relative to the susceptible wild-type, in herbicide untreated conditions. Fitness costs associated with herbicide resistance mutations are not universal and their expression depends on the particular mutation, genetic background, dominance of the fitness cost, and environmental conditions. The detrimental effects of herbicide resistance mutations on plant fitness may arise as a direct impact on fitness-related traits and/or coevolution with changes in other life history traits that ultimately may lead to fitness costs under particular ecological conditions. This brings the idea that a “lower adaptive value” of herbicide resistance mutations represents an opportunity for the design of resistance management practices that could minimize the evolution of herbicide resistance. It is evident that the challenge for weed management practices aiming to control, minimize, or even reverse the frequency of resistance mutations in the agricultural landscape is to “create” those agroecological conditions that could expose, exploit, and exacerbate those life history and/or fitness traits affecting the evolution of herbicide resistance mutations. Ideally, resistance management should implement a wide range of cultural practices leading to environmentally mediated fitness costs associated with herbicide resistance mutations.

## 1. Weeds in Agroecosystems

Agroecosystems are particular environments characterized by frequent, extensive, and intense disturbances and stress imposed by humans. As such, agroecosystems constitute an environment that selects for traits that maximise reproductive capacity, also called *r*- adaptive strategy (*r*, *per capita* rate of increase), in which high dispersion and growth rates, high resource allocation to reproduction, and a short life cycle are key to maximize plant fitness [1,2]. Adapted plants (i.e., weeds) of this particular disturbed and stressful environment embody the optimal phenotype and represent a major constraint to the quality and yield of grain crops [3] and agriculture sustainability [4].

Synthetic herbicides were developed and introduced into agroecosystems 70 years ago and continue today as the main agricultural tool to reduce weed densities securing global food production [5,6,7]. Both the global extension of agriculture frontiers and the substantial increase in herbicide reliance over the last decades combine to exert the strongest selection pressure ever experienced by weeds [8,9,10]. And this has inexorably led to herbicide resistance evolution in an ever-increasing list of weed species [11,12].

Herbicide resistance is the ultimate evidence of the extraordinary capacity of weeds to evolve under stressful conditions [9,11,13]. Herbicide resistance genes originate from random DNA mutations that endow a remarkable advantage to survive and reproduce, and therefore, are rapidly selected for and enriched in weed populations under herbicide treatment [14]. In particular, glyphosate resistance evolution has showed an alarming rate of increase among weeds in recent years [12].

## 2. Theoretical Considerations on Fitness Costs

Herbicide resistance is an adaptive evolutionary process in response to new environmental conditions (i.e., weed chemical control) in the agroecosystem. Herbicide resistance alleles are beneficial mutations that rapidly spread in weed populations under recurrent herbicide exposure [15,16]. These resistance mutations establish diverse defence mechanisms that protect plants from herbicide damage in different ways [11,17]. Some resistance mutations lead to amino acid substitutions in the herbicide target enzyme that change its configuration and geometry, altering distances to ligand H+ atoms and C- and N-terminal tails and water molecules [18,19,20]. These structural changes significantly reduce herbicide binding into the target enzyme, and thus confer resistance at the whole plant level (i.e., target site resistance mechanism). Alternatively, over-expression of the particular gene-encoding herbicide target enzyme increases its synthesis, which makes the herbicide insufficient to disrupt the normal plant metabolism [21]. Other mutations are responsible for regulating resistance mechanisms that minimize the amount of herbicide reaching the herbicide target site (i.e., non-target site resistance mechanism) [11,17]. For instance, enhanced herbicide metabolism (cytochrome P450 monooxygenases (CYP-450s), glutathione S-transferases (GSTs), or aldo-keto reductases (AKR)), reduced herbicide leaf uptake and translocation, and herbicide vacuolar sequestration are among the non-target site resistance mechanisms usually found in herbicide-resistant weeds [11,17,22]. However, whereas the biochemical basis associated with these non-target site resistance mechanisms has been elucidated, the molecular basis remains unknown.

Herbicide resistance mutations may pre-exist or arise spontaneously (de novo) within weed populations, and the rate at which they occur is very low [16,23,24], but see [25]. Despite the extraordinary plant fitness advantage endowed by herbicide resistance mutations in agroecosystems under herbicide selection, resistance mutations remain very rare traits in herbicide unselected weed populations [24,25,26].

A possible explanation for the low frequency of herbicide resistance alleles in unselected weed populations is their selective disadvantage imposed by associated fitness trade-offs. The prediction of a “lower adaptive value” or “deleterious effect” of resistance mutations in the original agroecosystem environment under no herbicide treatment would represent a cost of adaptation (i.e., fitness cost) which would limit the evolution of herbicide resistance by natural selection [27]. A fitness cost is the reduced success in contributing individuals to the next generation due to both or either impaired fecundity or survival [9,28]. After all, a fitness cost is the ultimate outcome of all genetic, biochemical, and physiological changes driven by a particular herbicide resistance mutation interacting within a particular genetic and ecological background [29].

Predicted fitness costs associated with resistance mutations have been a central paradigm in evolutionary ecology of herbicide resistance [15,28,30,31,32,33]. The fundamental evolutionary principle behind fitness costs is based on the resource-based allocation theory that predicts that plants divert resources into different functions to maximize their ecological success under the selection imposed by the environment [34,35,36,37]. As environmental resources are limited, any diversion of resources to one plant function would imply a decrease in allocation into other functions [36]. This theory underlies the trade-off usually found in plants between reproduction and defence functions [1,38,39]. It has been within this evolutionary context that herbicide resistance mutations, encoding for sophisticated defence mechanisms against herbicides, have been sought to divert resources and thus attract fitness costs [40].

Certainly, potential for allocation-based fitness costs in herbicide-resistant weeds correspond to resistance mutations responsible for herbicide metabolism via increased activity of endogenous detoxifying enzymes (e.g., cytochrome P450 monooxygenases) [41], reduced herbicide translocation within plants via vacuolar sequestration, or increased over-expression/duplication of herbicide resistance genes [11,21]. Provided that these herbicide resistance defence mechanisms require a diversion of resources to operate, it would be predictable the expression of associated fitness costs in plants carrying these resistance mechanisms. 

On the other hand, it would be less predictable the expression of allocation-based fitness costs associated with target site resistance mutations leading to changes in the structure and geometry of the herbicide target site enzyme due to changes in the amino acid sequence [29,32]. However, changes in catalytic activity, natural substrate affinity, and/or feedback inhibition of the mutated herbicide target site enzyme may alter normal plant metabolism, resulting in a whole plant fitness cost [29].

## 3. Fitness Costs Associated with Herbicide Resistance Mutations Are Not Universal

Numerous studies reviewing the existence of herbicide resistance fitness costs and their biochemical, molecular, physiological, and ecological mechanisms have been published elsewhere [28,29,30,31,32,42,43,44]. Despite a sound theoretical background, these studies have concluded that there is no universality in the expression of fitness costs associated with herbicide resistance mutations. Rather, these studies have determined that fitness cost expression in herbicide-resistant weeds depend on the particular herbicide resistance mutation [29,45,46,47,48], dominance of the fitness cost [49], genetic background [50], and environmental conditions [51,52].

An example of the complex biochemical, genetic, and environmental dependence of fitness costs is given by target site EPSPS mutations endowing resistance to glyphosate. A common DNA point mutation (*EPSPS* CCA to TCA) endowing moderate glyphosate resistance in several weed species leads the change of Pro to Ser (Pro-106-Ser) in the EPSPS enzyme [53]. This single amino acid substitution has been shown to not alter EPSPS kinetics and metabolism in *Eleusine indica*, rendering glyphosate resistant plants as fit as the glyphosate susceptible ones [54,55]. On the contrary, when another single EPSPS substitution (*EPSPS* ACT to ATT) replaces Thr for Ile (Thr-102-Ile) and combines in addition to the Pro-106-Ser EPSPS mutation (i.e., double EPSPS TIPS resistance mutation), two contrasting effects on *E. indica* resistant plants arise. Whereas the TIPS mutation shows a clear beneficial effect under glyphosate selection as the level of glyphosate resistance increases notoriously compared to the single Pro-106-Ser mutation, it shows a very high deleterious effect on plant fitness in environments under no glyphosate selection [54,55]. The fitness cost associated with the resistance TIPS mutation is only observed in homozygous resistant (RR) but not heterozygous resistant (RS) plants, and the magnitude of the cost increases significantly under interspecific plant resource competition [55]. The high fitness cost observed in plants with the homozygous TIPS mutations is likely due to the reduced EPSPS catalytic efficiency (Vmax), accumulation in excess of carbon-rich shikimate and quinate acids, and unbalanced polar metabolites from glycolysis and starch and sucrose metabolism [55].

## 4. Fitness Costs May Arise as Direct Effects of the Herbicide Resistance Mutations vs. Pleiotropic Effects on Other Plant Traits

The anticipated detrimental effects of herbicide resistance mutations on plant fitness may arise as a direct impact on fitness-related traits (e.g., reduced pollen viability) and/or co-evolution of loci interactions (e.g., resistance and non-resistance alleles) contributing to changes in other plant traits (e.g., seed dormancy) that ultimately may, in particular ecological environments, lead to fitness costs [15,44].

An example of a direct effect on fitness is the point mutation in the chloroplastic *psbA* gene, resulting in the amino acid substitution of serine to glycine (Ser-264-Gly) in the catalytic site of D1 protein. This mutation endows resistance to triazine herbicides, but also reduces the substrate affinity reducing the electron transfer rate in the photosystem II (PSII) (reviewed in [33,56,57]). This physiological change has been shown to decrease photosynthesis rate with direct negative effects on vegetative and reproductive growth rates in triazine resistant plants [33].

In other cases, herbicide resistance mutations have been shown to alter morphological, developmental, or phenological traits in weeds without necessarily a direct impact on plant fitness per se [58,59,60,61,62,63,64,65,66]. The changes in these traits are best thought of as changes in life history characters due to either subtle pleiotropic effects of resistance mutations or their coevolution with non-resistance life history traits in response to the wide range of selective factors operating in agroecosystems [9,67].

For instance, in some weedy grasses, particular ACCase resistance mutations have been shown to coevolve with higher levels of seed dormancy, absence of germination in dark conditions, and/or delayed seed germination (Table 1) [64,65,66]. Similarly, herbicide resistance mutations and mechanisms endowing resistance to different herbicide classes have been shown to covary with changes in plant size, root anatomy, leaf appearance rate, plant height, number of tillers, outcrossing vs. selfing mating rates, susceptibility to herbivory and diseases, and flowering time (Table 1) [58,60,61,62,68,69]. The coevolution of herbicide resistance and changes in life history traits is an adaptive response to the agroecosystem to maximize fitness of herbicide-resistant weed populations (i.e., population size, genetic diversity), and thus, the spread of herbicide resistance mutations [44].

Can we anticipate direct vs. indirect effects of herbicide resistance mutations on fitness? A number of traits, further from fitness (i.e., survival and fecundity) [28], related to development, phenology, metabolism, physiology, and morphology have been associated with a number of dissimilar herbicide resistance traits [59,60,66,70,71,72,73,74,75]. Inferences from these and other studies on the causal relationship between these life history and resistance traits are difficult to make. Are these modified life history traits a direct consequence of pleiotropic effects of herbicide resistance mutations, or the result of confounded effects driven by the presence of multiple resistance traits within populations, or local adaptation to particular environments which leads non-resistance loci to co-segregate with the resistance trait?

On the contrary, when herbicide resistance mutations drive changes in the architecture and structure of herbicide target enzymes, altering central kinetic parameters, direct detrimental effects on plant fitness are more likely to express and be predicted (reviewed in [29,32,45]). Changes in activity, substrate affinity, reaction speed, and/or feedback inhibition in herbicide target enzymes are strong predictors of the expression of fitness costs in herbicide-resistant plants. Similarly, energy constraints driven by herbicide resistance traits (e.g., gene amplification, enhanced metabolism) that are theoretically associated with higher cell energy budgets would also lead to the expression of direct fitness costs. However, current evidence suggests that estimation of cell energy budgets associated with these resistance mutations is necessary before any generalization.

## 5. Effects of Fitness Costs on the Equilibrium Frequency of Herbicide Resistance Mutations

In the agricultural landscape, there are dynamic, fluctuating, and diverse agroecological conditions imposed by the matrix of herbicide-treated and untreated areas. It is within these contrasting environments where the fitness of plants carrying herbicide mutations is defined and shaped by a suite of selection forces.

The beneficial effect of herbicide resistance mutations is realized in herbicide-treated areas due to the extraordinary survival advantage they confer, relative to the susceptible wild-type. Inevitably, herbicide resistance mutations will spread over time in continuously herbicide-treated environments. Fitness costs associated with a particular resistance mutation under particular ecological conditions, however, will disclose a deleterious effect as an adaptive disadvantage, relative to the susceptible wild-type, in the herbicide untreated area [15,42]. Thus, a resistance mutation expressing a fitness cost in a particular herbicide untreated ecological environment will exhibit limits to evolve by natural selection. Overall, the contrasting beneficial and deleterious effects of herbicide resistance mutations are the mechanisms that maintain resistance polymorphisms at the agricultural landscape scale with high and low frequencies of resistance mutations in herbicide-treated and untreated areas, respectively. As a result, fitness benefit and cost play a fundamental role in predicting the spread of herbicide resistance mutations and determining their equilibrium frequencies at the agricultural landscape level.

**Figure 1:**
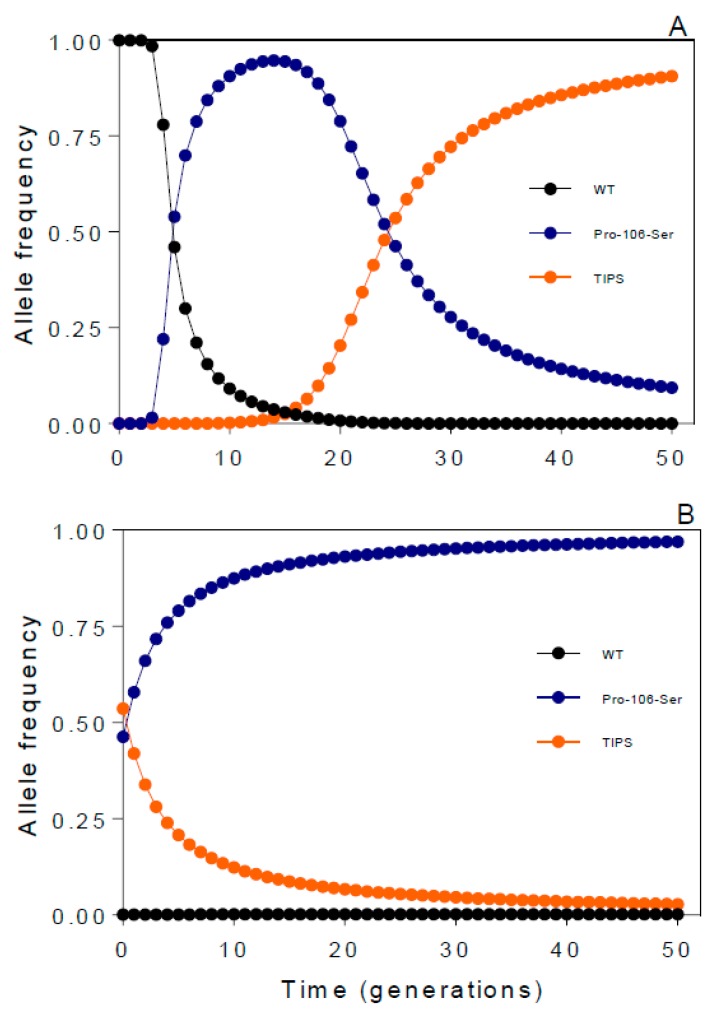
Predicted changes in the frequency of *Eleusine indica* EPSPS alleles (wild-type (WT), Pro-106-Ser, TIPS) over time (50 generations) in environments with (**A**) and without (**B**) glyphosate selection (1080 g ha^−1^). Simulation parameters are based on published [55] and unpublished studies. Input parameters in (**A**): Initial allele frequency (WT = 9.99999 × 10^−1^, Pro-106-Ser = 1.00 × 10^−6^, TIPS = 1.00 × 10^−10^); genotype fitness (WT/WT = 0.02, WT/Pro-106-Ser = 0.5, WT/TIPS = 0.6, Pro-106-Ser/Pro-106-Ser = 0.6, Pro-106-Ser/TIPS = 0.99, TIPS/TIPS = 0.99). Input parameters in (**B**): Initial allele frequency (WT = 9.26 × 10^−4^, Pro-106-Ser = 0.463, TIPS = 0.536); genotype fitness (WT/WT = 0.99, WT/Pro-106-Ser = 0.99, WT/TIPS = 0.99, Pro-106-Ser/Pro-106-Ser = 0.99, Pro-106-Ser/TIPS = 0.99, TIPS/TIPS = 0.30). Simulations were run for 50 generations using Populus software [82], assuming no further mutational events, genetic drift, and allele migration events.

The recent identification of a glyphosate resistance double mutation in *E. indica* can illustrate the impact of contrasting resistance benefits and costs associated with herbicide resistance mutations on their final equilibrium frequencies. Within a single *E. indica* population, it has been observed that individuals with the glyphosate resistance EPSPS Pro-106-Ser mutation coexist with plants which exhibit, in addition to the Pro-106-Ser mutation, a second EPSPS mutation, Thr-102-Ile—this double mutation is known as TIPS [54]. Under glyphosate selection, the single EPSPS Pro-106-Ser and TIPS mutations have been shown to endow, respectively, a moderate and high level of glyphosate resistance at both EPSPS and plant levels [54,76]. Remarkably, the high level of glyphosate resistance conferred by the EPSPS TIPS mutation is shared in both homozygous and heterozygous TIPS plants [76]. However, under no glyphosate selection, *E. indica* plants homozygous for the TIPS mutation pay an extremely high fitness cost which, interestingly, is not observed in plants heterozygous for the TIPS mutations, nor in individuals with the single EPSPS Pro-106-Ser mutation [55]. Thus, it may be predicted that from a very low allele frequency of 1 × 10^−10^ and after 50 generations under recurrent selection with recommended field glyphosate doses (1080 g ha^−1^), the frequency of the EPSPS TIPS allele will enrich and be nearly fixed (final freq. = 0.9) in the treated population at the expense of the wild-type (WT) (final freq. = 2.4 × 10^−9^) and Pro-106-Ser (final freq. = 0.09) alleles, which will become nearly extinct (Figure 1A). 

If the equilibrium allelic frequencies attained after 25 years of glyphosate use (Figure 1A) are considered as the starting point (WT = 9.26 × 10^−4^, Pro-106-Ser = 0.46, TIPS = 0.53) for an environment where glyphosate is discontinued for 50 generations, a basic simulation exercise predicts that, whereas the frequency of the WT allele will show a negligible increase (final freq. = 0.002), the frequency of the single Pro-106-Ser mutation will be nearly fixed (final freq. = 0.97) at the expense of the TIPS mutation (final freq. = 0.028) (Figure 1B). 

## 6. Implications of Fitness Costs to Resistance Management

The idea of a “lower adaptive value” of herbicide resistance mutations is often seen as an opportunity for the design of resistance management practices that could minimize the evolution of herbicide resistance [28,83,84]. However, a number of realizations need to be made to understand whether weed management practices can realistically exploit the predicted cost of adaptation of herbicide resistance mutations.

Firstly, excluding a very few exceptions (see [29,48,55,56,62]), fitness costs, regardless of whether they arise as a direct effect of the resistance mutation vs. their coevolution with changes in life history traits, are environmentally dependent, meaning that they will solely express under certain ecological conditions. They may not always express, as either compensatory molecular evolution of costs is possible [85] or the “right” ecological conditions to bring the resistance mutation at disadvantage may not be present.

Secondly, the detrimental effect of fitness costs on plants carrying the resistance mutation, relative to the susceptible wild-type, is immediately masked under conditions of continuous herbicide treatment. It is straightforward then that for a management practice to exploit a fitness cost of a resistance mutation, no herbicide treatments need to be considered during successive growing seasons (i.e., herbicide “off” years), single entire season (i.e., herbicide “off” and “on” years) or, at least, a limited time window within a growing season (e.g., non-chemical fallow). The final impact of removing the herbicide selective benefit on the frequency of the resistance mutations at the landscape level will be a function of the magnitude of the fitness cost (negligible, moderate, high), period of time (single vs. several generations/growing seasons) under no herbicide use, and area covered by the resistant population (single field vs. farm vs. regional area).

It is evident that the challenge for weed management practices aiming to control, minimize, or even reverse the frequency of resistance mutations in the agricultural landscape is to “create” those agroecological conditions that could expose, exploit, and exacerbate those life history and/or fitness traits affecting the evolution of herbicide resistance mutations [9,67]. Ideally, resistance management should implement a wide range of practices leading to environmentally mediated fitness costs associated with herbicide resistance mutations.

Generation of dynamic spatial and temporal diverse agroecological conditions are possible through the implementation of management practices, such as the use of grazed and ungrazed pasture phases [86], cover crops [87], choice of competitive cultivars [88], changes in cultural practices such as seeding and harvest time, crop row spacing, density and orientation [89], implementation of soil tillage [83], and management of fence lines and field margins [90], just to mention a few. These diverse agroecological conditions imply environmental changes in fluctuating temperatures and light intensity and quality (red/far red ratio) at soil levels and under crop canopies and demand of plant resources together, which in turn may bring changes in soil chemical and physical properties. More diversified agricultural landscapes will likely select against herbicide resistance mutations through exploitation of fitness costs compared to agroecosystems with simplified weed management practices.

## Figures and Tables

**Table 1:** Examples of herbicide-resistant weeds where resistance mutations have been associated with decreased fitness and/or altered life history traits.

Resistance Mutation/Trait	Weed Species	Fitness/Life History Trait	Environment	Biochemical/Physiological Change	Reference
ACCase/ALS CYP-450 metabolism	*Lolium rigidum*	Reduced RGR *, fecundity	Crop competition		[58,77]
ACCase/ALS target site resistance and CYP-450 metabolism	*L. rigidum*	Higher seed dormancy	Controlled conditions		[66]
*EPSPS* over-expression	*L. perenne*	Reduced height, leaf area, fecundity	Intra-specific competition in rain fed conditions		[78]
*EPSPS* TIPS mutation	*Eleusine indica*	Reduced RGR, fecundity	Crop competition	Reduced EPSPS Vmax Altered C-rich metabolite levels	[55]
*ACCase* Ile-1781-Leu	*L. rigidum*	Light requirement for seed germination	Controlled conditions	Changes in sensitivity of phytochrome B	[64] (Vila-Aiub et al. unpublished)
*ACCase* 2078	*Alopecurus myosuroides*	Lower germination rate	Wheat competition	Reduced ACCase activity	[48]
*psbA* Ser-264-Gly	Many broadleaf species	Reduced RGR, fecundity	Controlled and field conditions	Reduced QB affinity, inefficient PSII electron transport, lower photosynthesis	Reviewed in [33]
*psbA* Ser-264-Gly	*Amaranthus powelii*	Higher susceptibility to herbivory	Field conditions	Higher leaf N concentration	[68,79]
*ALS* Trp-574-Leu	*A. powelli*	Smaller roots, reduced leaf area and RGR	Intra-specific competition	Likely impaired ALS function	[62]
Glyphosate resistance	*Ipomoea purpurea*	Higher selfing rate	Controlled and field conditions	Lower anther–stigma distance	[61]
*EPSPS* amplification	*Kochia scoparia*	Delayed flowering	Controlled conditions		[71]
*AUX/IAA KsIAA16* *Gly-73-Asn*	*K. scoparia*	Reduced RGR, leaf area, height, fecundity	Controlled conditions		[80,81]

* RGR: Relative growth rate.

## References

[1] Grime J.P. (1977). Evidence for the existence of three primary strategies in plants and its relevance to ecological and evolutionary theory. Am. Nat..

[2] MacArthur R.H., Wilson E.O. (1967). The Theory of Island Biogeography.

[3] Cousens R., Mortimer M. (1995). Dynamics of Weed Populations.

[4] Oerke E.C. (2006). Crop losses to pests. J. Agric. Sci..

[5] Powles S. (2014). Global Herbicide Resistance Challenge. Pest Manag. Sci..

[6] Powles S.B. (2008). Evolved glyphosate-resistant weeds around the world: Lessons to be learnt. Pest Manag. Sci..

[7] National Research Council (2000). The Future Role of Pesticides in US Agriculture.

[8] Palumbi S.R. (2001). Evolution-Humans as the world’s greatest evolutionary force. Science.

[9] Neve P., Vila-Aiub M., Roux F. (2009). Evolutionary-thinking in agricultural weed management. New Phytol..

[10] James C. (2016). Executive Summary of Global Status of Commercialized Biotech/GM Crops.

[11] Powles S.B., Yu Q. (2010). Evolution in action: Plants resistant to herbicides. Ann. Rev. Plant Biol..

[12] Heap I. The International Survey of Herbicide Resistant Weeds. www.weedscience.com.

[13] Neve P., Busi R., Renton M., Vila-Aiub M.M. (2014). Expanding the eco-evolutionary context of herbicide resistance research. Pest Manag. Sci..

[14] Maxwell B.D., Mortimer A.M., Powles S.B., Holtum J.A.M. (1994). Selection for Herbicide Resistance. Herbicide Resistance in Plants: Biology and Biochemistry.

[15] Lenormand T., Harmand N., Gallet R. (2018). Cost of resistance: An unreasonably expensive concept. Rethink. Ecol..

[16] Gressel J., Levy A.A. (2006). Agriculture: The selector of improbable mutations. Proc. Natl. Acad. Sci. USA.

[17] Délye C., Jasieniuk M., Le Corre V. (2013). Deciphering the evolution of herbicide resistance in weeds. Trends Genet..

[18] McCourt J.A., Pang S.S., King-Scott J., Guddat L.W., Duggleby R.G. (2006). Herbicide-binding sites revealed in the structure of plant acetohydroxyacid synthase. Proc. Natl. Acad. Sci. USA.

[19] Schönbrunn E., Eschenburg S., Shuttleworth W.A., Schloss J.V., Amrhein N., Evans J.N., Kabsch W. (2001). Interaction of the herbicide glyphosate with its target enzyme 5-enolpyruvylshikimate 3-phosphate synthase in atomic detail. Proc. Natl. Acad. Sci. USA.

[20] Zhang H., Tweel B., Tong L. (2004). Molecular basis for the inhibition of the carboxyltransferase domain of acetyl-coenzyme-A carboxylase by haloxyfop and diclofop. Proc. Natl. Acad. Sci. USA.

[21] Gaines T.A., Patterson E.L., Neve P. (2019). Molecular mechanisms of adaptive evolution revealed by global selection for glyphosate resistance. New Phytol..

[22] Pan L., Yu Q., Han H., Mao L., Nyporko A., Fan L., Powles S.B. (2019). Aldo-keto reductase metabolizes glyphosate and confers glyphosate resistance in Echinochloa colona. Plant Physiol..

[23] Casale F.A., Giacomini D.A., Tranel P.J. (2019). Empirical investigation of mutation rate for herbicide resistance. Weed Sci..

[24] Busi R., Gaines T.A., Walsh M.J., Powles S.B. (2012). Understanding the potential for resistance evolution to the new herbicide pyroxasulfone: Field selection at high doses versus recurrent selection at low doses. Weed Res..

[25] Neve P., Powles S. (2005). High survival frequencies at low herbicide use rates in populations of Lolium rigidum result in rapid evolution of herbicide resistance. Heredity.

[26] Preston C., Powles S.B. (2002). Evolution of herbicide resistance in weeds: Initial frequency of target site-based resistance to acetolactate synthase-inhibiting herbicides in Lolium rigidum. Heredity.

[27] Fisher R.A. (1958). The Genetical Theory of Natural Selection.

[28] Cousens R.D., Fournier-Level A. (2018). Herbicide resistance costs: What are we actually measuring and why?. Pest Manag. Sci..

[29] Vila-Aiub M.M., Yu Q., Powles S.B. (2019). Do plants pay a fitness cost to be resistant to glyphosate?. New Phytol..

[30] Bergelson J., Purrington C.B. (1996). Surveying patterns in the cost of resistance in plants. Am. Nat..

[31] Vila-Aiub M.M., Neve P., Roux F. (2011). A unified approach to the estimation and interpretation of resistance costs in plants. Heredity.

[32] Vila-Aiub M.M., Neve P., Powles S.B. (2009). Fitness costs associated with evolved herbicide resistance alleles in plants. New Phytol..

[33] Holt J.S., Thill D.C., Powles S.B., Holtum J.A.M. (1994). Growth and Productivity of Resistant Plants. Herbicide Resistance in Plants. Biology and Biochemistry.

[34] Harper J. (1977). Population Biology of Plants.

[35] Solbrig O.T., Schulze E.D., Mooney H.A. (1994). Plant Traits and Adaptive Strategies: Their Role in Ecosystem Function. Biodiversity and Ecosystem Function.

[36] Lerdau M., Gershenzon J., Bazzaz F., Grace J. (1997). Allocation Theory and Chemical Defense. Plant Resource Allocation.

[37] Strauss S.Y., Rudgers J.A., Lau J.A., Irwin R.E. (2002). Direct and ecological costs of resistance to herbivory. Trends Ecol. Evol..

[38] Coley P.D., Bryant J.P., Chapin F.S. (1985). Resource availability and plant antiherbivore defense. Science.

[39] Chapin F.S., Autumn K., Pugnaire F. (1993). Evolution of suites of traits in response to environmental-stress. Am. Nat..

[40] Herms D.A., Mattson W.J. (1992). The dilemma of plants—To grow or defend. Q. Rev. Biol..

[41] Yu Q., Powles S. (2014). Metabolism-based herbicide resistance and cross-resistance in crop weeds: A threat to herbicide sustainability and global crop production. Plant Physiol..

[42] Vila-Aiub M.M., Gundel P.E., Preston C. (2015). Experimental methods for estimation of plant fitness costs associated with herbicide-resistance genes. Weed Sci..

[43] Keshtkar E., Abdolshahi R., Sasanfar H., Zand E., Beffa R., Dayan F.E., Kudsk P. (2019). Assessing Fitness Costs from a Herbicide-Resistance Management Perspective: A Review and Insight. Weed Sci..

[44] Baucom R.G. (2019). Evolutionary and ecological insights from herbicide resistant weeds: What have we learned about plant adaptation, and what is left to uncover?. New Phytol..

[45] Yu Q., Han H., Vila-Aiub M.M., Powles S.B. (2010). AHAS herbicide resistance endowing mutations: Effect on AHAS functionality and plant growth. J. Exp. Bot..

[46] Ashigh J., Tardif F. (2007). An Ala_205_Val substitution in acetohydroxyacid synthase of Eastern black nightshade (Solanum ptychanthum) reduces sensitivity to herbicides and feedback inhibition. Weed Sci..

[47] Purrington C.B., Bergelson J. (1999). Exploring the physiological basis of costs of herbicide resistance in Arabidopsis thaliana. Am. Nat..

[48] Menchari Y., Chauvel B., Darmency H., Délye C. (2008). Fitness costs associated with three mutant acetyl-coenzyme A carboxylase alleles endowing herbicide resistance in black-grass Alopecurus myosuroides. J. Appl. Ecol..

[49] Roux F., Gasquez J., Reboud X. (2004). The dominance of the herbicide resistance cost in several Arabidopsis thaliana mutant lines. Genetics.

[50] Paris M., Roux F., Berard A., Reboud X. (2008). The effects of the genetic background on herbicide resistance fitness cost and its associated dominance in Arabidopsis thaliana. Heredity.

[51] Frenkel E., Matzrafi M., Rubin B., Peleg Z. (2017). Effects of environmental conditions on the fitness penalty in herbicide resistant brachypodium hybridum. Front. Plant Sci..

[52] Williams M.M.I., Jordan N., Yerkes C. (1995). The fitness cost of triazine resistance in jimsonweed (Datura stramonium L.). Am. Midl. Nat..

[53] Sammons R.D., Gaines T.A. (2014). Glyphosate resistance: State of knowledge. Pest Manag. Sci..

[54] Yu Q., Jalaludin A., Han H., Chen M., Sammons R.D., Powles S.B. (2015). Evolution of a double amino acid substitution in the EPSP synthase in Eleusine indica conferring high level glyphosate resistance. Plant Physiol..

[55] Han H., Vila-Aiub M.M., Jalaludin A., Yu Q., Powles S.B. (2017). A double EPSPS gene mutation endowing glyphosate resistance shows a remarkably high resistance cost. Plant Cell Environ..

[56] Gronwald J.W., Powles S.B., Holtum J.A.M. (1994). Resistance to Photosystem II Inhibiting Herbicides. Herbicide Resistance in Plants. Biology and Biochemistry.

[57] Devine M.D., Shukla A. (2000). Altered target sites as a mechanism of herbicide resistance. Crop Prot..

[58] Vila-Aiub M.M., Neve P., Powles S.B. (2005). Resistance cost of a cytochrome P450 herbicide metabolism mechanism but not an ACCase target site mutation in a multiple resistant Lolium rigidum population. New Phytol..

[59] Bravo W., Leon R.G., Ferrell J.A., Mulvaney M.J., Wood C.W. (2017). Differentiation of life-history traits among Palmer amaranth populations (Amaranthus palmeri) and its relation to cropping systems and glyphosate sensitivity. Weed Sci..

[60] Van Etten M.L., Kuester A., Chang S.M., Baucom R.S. (2016). Fitness costs of herbicide resistance across natural populations of the common morning glory, Ipomoea purpurea. Evolution.

[61] Kuester A., Fall E., Chang S.M., Baucom R.S. (2017). Shifts in outcrossing rates and changes to floral traits are associated with the evolution of herbicide resistance in the common morning glory. Ecol. Lett..

[62] Tardif F.J., Rajcan I., Costea M. (2006). A mutation in the herbicide target site acetohydroxyacid synthase produces morphological and structural alterations and reduces fitness in Amaranthus powellii. New Phytol..

[63] Comont D., Knight C., Crook L., Hull R., Beffa R., Neve P. (2019). Alterations in Life-History Associated With Non-target-site Herbicide Resistance in Alopecurus myosuroides. Front. Plant Sci..

[64] Vila-Aiub M.M., Neve P., Steadman K.J., Powles S.B. (2005). Ecological fitness of a multiple herbicide-resistant Lolium rigidum population: Dynamics of seed germination and seedling emergence of resistant and susceptible phenotypes. J. Appl. Ecol..

[65] Délye C., Menchari Y., Michel S., Cadet É., Le Corre V. (2013). A new insight into arable weed adaptive evolution: Mutations endowing herbicide resistance also affect germination dynamics and seedling emergence. Ann. Bot..

[66] Owen M.J., Michael P.J., Renton M., Steadman K.J., Powles S.B. (2011). Towards large-scale prediction of Lolium rigidum emergence. II. Correlation between dormancy and herbicide resistance levels suggests an impact of cropping systems. Weed Res..

[67] Bagavathiannan M.V., Davis A.S. (2018). An ecological perspective on managing weeds during the great selection for herbicide resistance. Pest Manag. Sci..

[68] Gassmann A.J. (2005). Resistance to herbicide and susceptibility to herbivores: Environmental variation in the magnitude of an ecological trade-off. Oecologia.

[69] Salzmann D., Handley R.J., Mueller-Scharer H. (2008). Functional significance of triazine-herbicide resistance in defence of Senecio vulgaris against a rust fungus. Basic Appl. Ecol..

[70] Mithila J., McLean M.D., Chen S., Christopher Hall J. (2012). Development of near-isogenic lines and identification of markers linked to auxinic herbicide resistance in wild mustard (Sinapis arvensis L.). Pest Manag. Sci..

[71] Martin S.L., Benedict L., Sauder C.A., Wei W., da Costa L.O., Hall L.M., Beckie H.J. (2017). Glyphosate resistance reduces kochia fitness: Comparison of segregating resistant and susceptible F2 populations. Plant Sci..

[72] Kumar V., Jha P., Lim C.A., Stahlman P.W. (2018). Differential Germination Characteristics of Dicamba-Resistant Kochia (Bassia scoparia) Populations in Response to Temperature. Weed Sci..

[73] Osipitan O.A., Dille J.A. (2017). Fitness Outcomes Related to Glyphosate Resistance in Kochia (Kochia scoparia): What Life History Stage to Examine?. Front. Plant Sci..

[74] Beckie H.J., Blackshaw R.E., Leeson J.Y., Stahlman P.W., Gaines T.A., Johnson E.N. (2018). Seedbank persistence, germination and early growth of glyphosate-resistant Kochia scoparia. Weed Res..

[75] Baucom R.S., Mauricio R. (2004). Fitness costs and benefits of novel herbicide tolerance in a noxious weed. Proc. Natl. Acad. Sci. USA.

[76] Vila-Aiub M., García F., Han H., Jalaludin A., Yu Q., Powles S.B. (2019). Resistance Benefit Endowed by a Double EPSPS Glyphosate Resistance Mutation (TIPS) in Eleusine Indica. Resistance 2019.

[77] Vila-Aiub M.M., Neve P., Powles S.B. (2009). Evidence for an ecological cost of enhanced herbicide metabolism in Lolium rigidum. J. Ecol..

[78] Yanniccari M., Vila-Aiub M., Istilart C., Acciaresi H., Castro A.M. (2016). Glyphosate resistance in perennial ryegrass (Lolium perenne L.) is associated with a fitness penalty. Weed Sci..

[79] Gassmann A.J., Futuyma D.J. (2005). Consequence of herbivory for the fitness cost of herbicide resistance: Photosynthetic variation in the context of plant-herbivore interactions. J. Evol. Biol..

[80] Murphy B.P., Tranel P.J. (2019). Target-Site Mutations Conferring Herbicide Resistance. Plants.

[81] LeClere S., Wu C., Westra P., Sammons R.D. (2018). Cross-resistance to dicamba, 2, 4-D, and fluroxypyr in Kochia scoparia is endowed by a mutation in an AUX/IAA gene. Proc. Natl. Acad. Sci. USA.

[82] Alstad D. (2001). Basic Populus Models of Ecology.

[83] Colbach N., Chauvel B., Darmency H., Délye C., Le Corre V. (2016). Choosing the best cropping systems to target pleiotropic effects when managing single-gene herbicide resistance in grass weeds. A blackgrass simulation study. Pest Manag. Sci..

[84] Jordan N., Kelrick M., Brooks J., Kinerk W. (1999). Biorational management tactics to select against triazine-resistant Amaranthus hybridus: A field trial. J. Appl. Ecol..

[85] Uyenoyama M., Glass E. (1986). Pleiotropy and the Evolution of Genetic Systems Conferring Resistance to Pesticides. Pesticide Resistance. Strategies and Tactics for Management.

[86] Doole G.J. (2008). Optimal management of annual ryegrass (Lolium rigidum Gaud.) in phase rotations in the Western Australian Wheatbelt. Aust. J. Agric. Resour. Econ..

[87] Osipitan O.A., Dille J.A., Assefa Y., Radicetti E., Ayeni A., Knezevic S.Z. (2019). Impact of Cover Crop Management on Level of Weed Suppression: A Meta-Analysis. Crop Sci..

[88] Sadras V.O., Lawson C. (2011). Genetic gain in yield and associated changes in phenotype, trait plasticity and competitive ability of South Australian wheat varieties released between 1958 and 2007. Crop Pasture Sci..

[89] Borger C.P., Hashem A., Pathan S. (2010). Manipulating crop row orientation to suppress weeds and increase crop yield. Weed Sci..

[90] Maino J.L., Renton M., Hoffmann A.A., Umina P.A. (2019). Field margins provide a refuge for pest genes beneficial to resistance management. J. Pest Sci..

